# Remedies Affecting the Mammæ

**Published:** 1889-07

**Authors:** Wm. Jefferson Guernsey

**Affiliations:** 4430 Frankford Avenue, Philadelphia, Pa.


### REMEDIES

### In General Affecting the

Mammæ.—*Acon.* Æscul. Æthus. Agar. AGNUS. All-sat. Alum. Ambr. Am-cb. Am-mur. Anac. Angus. Ant-cr. Ant-tar. Apis. Arg-nit. ARN. *Arsen.* Arum. Asaf. Bary-cb. BELL. Berber. *Borax.* *Bovist.* Brom. BRYON. Cactus. CALC. *Calc-ph.* Calad. Camph. Can-sat. Canth. CARB-AN. CARB-VEG. Castor. Caust. CHAM. Chel. China. *Cicut.* Cimicif. Cina. Cistus. CLEM. Cocul. Coff. Colo. CON. *Crot-tig.* Curare. Cycla. Dig. DULC. Fragar. Gamb. Gels. GRAPH. Gratiol. Guaiac. Ham. *Hep.* Ign. Ipec. *Iod.* Kali-bi. Kali-cb. Kreos. Lach. LAC-CAN. Lac-defl. Lactuc. Lauro. Ledum. Lepi. *Lil-tig.* Lyc. Mag-cb. Mangan. Merc-cor. *Merc-sol.* *Merc-viv.* Mezer. Millef. Mosch. Murex. Na-cb. Na-mur. Niccol. Nit-ac. Nux-jug. Nux-vom. Opi. Petrol. *Phellan.* PHOS. Phos-ac. PHYTOL. Plat. Plumb. Prunus. Psorn. PULS. Ran-bul. Ran-scel. Raphan. Ratan. *Rheum.* Rhod. *Rhus.* Ruta. Sabad. Sabina. Samb. Sang Sars. Secale. *Sepia.* SIL. Spong. Squil. Stan. Staph. Stram. *Sul.* Tarent. Thu. *Uva-u.* Verat. *Zinc.*

Mammæ, Left.—Æthus. Agar. Alum. Ambr. Apis. Berber. *Borax.* *Bovist.* Cactus. Calc. Calc-ph. Cistus. CON. Cycla. Gratiol. Lac-can. *Lil-tig.* Lyc. Mag-cb. Mosch. *Phellan.* Phos. *Phytol.* Plumb. SIL. Spong. Zinc.

Mammæ, Right.—All-sat. Ambr. Calc. CON. Gamb. Gratiol. Kali-bi. Kreos. Lac-can. Mezer. PHYTOL. Plumb. Psorn. Sang. SIL. Zinc.

### SUBJECTIVE SYMPTOMS.

Aching.—Apis. *Bovist.* Con. LAC-CAN. Lil-tig. Mosch. Stram. Zinc.

Air, streaming through.—Cycla.

Burning.—Æscul. Ambr. Apis. Arsen. Bell. Calc-ph. Con. Iod. Laur. Led. Lyc. Phos. Sang.

Coldness.—Cimicif. Coccul. Dig. Rhus.

Compression.—Thu.

Compression Backward.—Thu.

Constriction.—Lil-tig. Sang.

Contraction.—*Borax.* Calc-ph. Stram. Verat.

Cramp-like pain.—*Lil-tig.* Plat.

Cutting.—Bell. Lach. Lepi. Lil-tig.

Darting.—Carb-an. Gratiol. Iod. Kali-bi.

Drawing.—Calc-ph. Kreos. Lil-tig.

Fullness.—Bell. BRY. Clem. Cycla. LAC-CAN. Lactuc. Merc-v. Nux. PHYTOL. Secale. Sep.

Grasping.—Lil-tig.

Griping.—Bovis.

Gurgling.—Crot-tig.

Heaviness.—Bell. Bry. Clem. Lil-tig. Thu.

Itching.—Agar. Alum. Anac. Ant-cr. Arn. Ars. Bary-cb. Berber. Bovist. Calc. Canth. Carb-veg. Caust. Con. Kali-cb. Ledum. Lyc. Mezer. Na-mur. Niccol. Nux-jug. Phellan. Phos. Plumb. Rhus. Sabad. Sep. Spong. Squil. Stan. Staph. Sul.

Lancination.—See Cutting.

Milk flowing in, as from.—Kreos.

Pain (undefined).—Ang. Ant-cr. Arn. Bary-cb. *Bell.* Borax. Bry. Cactus. Calad. CALC. *Con.* Crot-tig. Cycla. Iod. Kali-bi. Lach. LAC-CAN. Laur. *Lil-tig.* Merc-sol. Murex. Phel. Phos. Rheum. Rhus. Sang. SIL. Verat. Zinc.

Pain extending backward (through chest, to lumbar region, to scapula, to spine).—Lil-tig.

Pain extending downward to navel.—Agar.

Pain extending downward to sides.—Prunus.

PAIN EXTENDING FORWARD to beneath sternum.—Sang.
PAIN EXTENDING INWARD.—*Phel.*
PAIN EXTENDING NIPPLE (from periphery to the).—Kreos.
PAIN EXTENDING OUTWARD.—Gels. Mezer.
PAIN EXTENDING UPWARD to ARMS.—Curare.
PAIN EXTENDING UPWARD to NECK.—Lil-tig.
PAIN EXTENDING UPWARD to SHOULDERS.—Lil-tig. Mag-cb.
PAIN, LABOR, as though from.—Lach.
PINCHING.—Agar. Calc-ph.
PRESSURE.—Am-m. Calc-ph. Phos. Phos-ac.
PRESSURE, ACUTE.—Phos-ac.
PRICKLING.—Calc. Cimic. Ran-scel.
PULSATION.—Bell.
RAWNESS.—Merc-v.
SENSITIVENESS.—See TENDERNESS.
SHIVERING, as if.—Guaiac. Nux. Petrol.
SHOOTING.—Calc-ph.
SORENESS.—All-sat. *Angust.* Ambr. ARN. Arum. Bry. Calad. *Calc. Calc-ph. Cicut.* Graph. LAC-CAN. Merc-v. Na-mur. *Phytol.* Rhod. Sang. Sep. Sil.
STITCHES. — Æthus. All-sat. Alum. Ambr. Apis. Arg-nit. Bary-cb. Berber. *Borax.* Bry. Calc. Carb-an. Cimicif. Clem. CON. Cycla. Gamb. Gels. Graph. Gratiol. Ign. Iod. Kali-bi. Kali-cb. Kreos. Laur. Lil-tig. Lyc. Mag-cb. Mezer. Murex. Na-mur. Phel. *Phos.* Plumb. Prunus. Psorn. Rheum. Sang. *Sep.* Sil. Thu. Zinc.
STITCHES, FINE.—Plumb.
SUPPURATIVE PAIN. — CALC. Clem. Hep. Phos. Plumb. Sil.
SUPPURATIVE SENSATION.—SIL.
SWELLING, as if.—Berber.
TEARING.—Am-cb. Am-m. Bary-cb. Calc. Calc-ph. Carb-veg. Con. Crot-tig. Gratiol. Kali-cb. Sang.
TENDERNESS.—*Calc.* Cham. Clem. *Con.* Graph. LAC-CAN. Merc-v. Na-mur. *Phytol.* Thu. Zinc.
TENSION.—Cycla. Puls.
TINGLING.—Sabin.
UNPLEASANT (indescribable).—*Phos.*

### OBJECTIVE SYMPTOMS.

ABSCESS.—See SUPPURATION.
ATROPHY.—Arsen. *Con.* Mag. *Iod.* Kali-iod. Kreos. Nit-ac. Nux-mos. Sarsap.

BLUISH, LIVID hue.—Lach. Phos. Plumb.
BLUISH, RED hue.—Kreos.
DISTENSION.—Cycla. Zinc.
EMACIATION.—See ATROPHY.
FEVER (milk fever).—*Acon.* ARN. BELL. BRY. Cham. Coff. Ign. Merc-v. Opi. *Rhus.*
FLABBINESS.—Bell. Camph. Con. Iod.
HEAT in.—Acon. Bell. Bry. Calc-ph. Mangan. Raphan. *Sul.*
INDURATION.—Arn. BELL. Bry. Calc. Calc-ph. CARB-AN. CHAM. CLEM. Coloc. CON. Cycla. *Graph.* Ham. LAC-CAN. Lepi. Lyc. Merc-v. Nit-ac. Phos. PHYT. Plumb. Puls. Ruta. Sep. SIL. Spong. *Sul.*
INFLAMMATION. — Bell. Bry. Calc. CARB-AN. CARB-VEG. Cistus. Con. Hep. Lac-can. Merc-v. Phos. *Phyt.* Sil. Sul.
MILK BAD tasting.—Borax. Merc-v.
MILK BITTER tasting.—Rheum.
MILK BLUISH.—Lach.
MILK CHEESY.—Cham.
MILK COPIOUS (too).—*Acon.* Ant-tart. Asaf. Bell. Borax. BRYON. *Calc.* China. Con. Iod. Kreos. Lach. *Lac-can.* Lyc. Nux-vom. Phos. PHYTOL. PULS. Rhus. Stan. Staph. Stram.
MILK PURULENT.—Cham.
MILK RETARDED by cicatrices.—GRAPH. *Phyt.*
MILK SALT tasting.—Carb-an.
MILK SCANT.—AGNUS. Asaf. Bell. Bry. CALC. Caust. Cham. Chel. China. DULC. *Lac-can.* Lyc. Merc-v. Millef. *Phel.* Phos. *Puls.* Rhus. Samb. Secale. Sep. Sul. *Uva-u. Zinc.*
MILK SPOILED.—Bell. *Borax.* Carb-an. CHAM. Cina. Ipec. Lach. *Merc.* Nux. Puls. *Rheum.* Samb. Stan.
MILK STRINGY.—Kali-bi.
MILK THICK.—Borax.
MILK THIN.—Carb-an. Kali-bi. Lach.
MILK WANTING.—Agnus. Asaf. Lac-can. Urt-u.
MILK YELLOW.—Rheum.
REDNESS, RADIATING from centre.—Bell. Sul.
REDNESS, STREAKS of.—Phos. Rhus.
SUPPURATION, INEVITABLE.—HEP. *Sil.*
SUPPURATION, THREATENED.—Asaf. Bell. Calc. Cistus. Dulc. Kali-cb. Kreos. Lac-can. Merc-v. Na-cb. Phos. PHYTOL. Puls. Sep. Sul.
SWELLING.—Æthus. All-sat. Apis. Asaf. *Bell.* Berb. Brom. *Bry. Calc.* Cham. Clem. *Con.* Cycla. Dulc. Graph. Hep. Lach. LAC-CAN. Lyc. Merc-cor. *Merc-sol.* Merc-v. Phos. PHYTOL.

Plumb. Puls. Ratan. Sabina. Samb. SIL. Sul. Tarent. *Uva-u.* Zinc.

SWELLING LUMPS like marbles.—*Lac-can.* Phytol.

ULCERATION.—Phos. Phyt. Sil. Sul.

ULCERATION FISTULOUS.—Phos. Phyt. Sil.

### AGGRAVATIONS AND AMELIORATIONS.

AFTERNOON, AGG.—Æth. Bell. Bry. Nit-ac. Phos. Puls. Sang.

ASCENDING stairs, AGG.—Bell. Calc. Carb-an. LAC-CAN. Lyc. Nit-ac. Phos.

BED (in), AGG.—Murex.

BENDING FORWARD, AGG.—Gratiol.

BREATHING in.—See INSPIRING.

COLD, AGG. from.—Sep.

COLD, TAKING, AGG.—*Acon.* Bell. Bry. Cact. Calc. Cham. DULC. Merc. Nux. Phos. Puls. Rhus.

CONTUSION, AGG.—*Arn.* Carb-an. CON. Ham.

DAY, AGG.—Con.

EMPTY, AGG. when.—*Bov.*

ERECT, AGG. on becoming.—Graph.

EVENING, AGG.—Arn. Bell. Bry. *Con.* LAC-CAN. Nit-ac. Phos. Puls. Spong.

EXERCISING, AGG.—Angus. Laur. Ran-bul.

EXERCISING ARMS, AGG.—Angus. Ant-cr.

EXERCISING, OPEN AIR, AGG.—Am-m.

FLOW OF MILK, AMEL.—Cycla.

HOLDING THE.—See SUPPORTING.

INSPIRING, AGG.—Carb-an. Gratiol. Lac-can. Mag-cb. Plumb. Prun.

INSPIRING, DEEPLY, AGG.—Prunus. Sang.

JAR, AGG.—BELL. Calc. Carb-an. LAC-CAN. Lyc. Nit-ac. Phos.

LIFTING.—See SUPPORTING.

LYING ON LEFT SIDE, AGG.—Lil-tig.

LYING ON PAINFUL SIDE, AGG.—Lil-tig.

MENSES, AGG. AFTER.—Cycla. Thu.

MENSES, AGG. BEFORE.—*Calc. Con.* Cycla. *Lac-can.* Sang. Spong.

MENSES, AGG. DELAYED.—Bry-cb. *Calc. Con.* Dulc. Iod. Merc-v. *Phos.* Rhus. Thu. Zinc.

MENSES AGG. DURING.—Calc. Carb-an. Caust. *Con.* Dulc. Iod. Lac-can. Lac-defl. Merc-v. *Phos.* Sang. Thu. Zinc.

MENSES AGG. SUPPRESSED.—Ratan.
MORNING, AGG.—Calad. Calc. Carb-v. Chel. *Lil-tig.* Nux-v. Rhus. Sang. Zinc.
MORNING, AMEL.—Spong.
MORNING, BED, AGG. in.—Plumb.
MOTION, AGG.—Sep.
NIGHT, AGG.—Acon. Arn. Ars. Cham. Con. Dulc. Graph. Hep. Iod. Merc-v. Nit-ac. Plumb. Sil.
NOON, AGG.—Mag-cb.
NURSING, AGG.—Borax. Carb-an. *Crot-tig.* Kali-cb. Phel.
NURSING OPPOSITE BREAST, AGG.—*Borax.*
PAROXYSMALLY, AGG.—Castor.
PERIODICALLY, AGG.—Ars. Kreos. Merc-sol.
POSITION AND CHANGE OF.—Lit-tig.
PRESSURE, AGG.—Ant-cr. *Calc.* Carb-v. LAC-CAN. Merc-v. Murex.
PRESSURE, AMEL.—Kreos.
REST, AGG.—Rhus.
RIDING, AGG.—SEP.
RUBBING, AGG.—Con.
RUBBING, AMEL.—Castor.
SITTING, AGG.—Prun. Thu.
SNEEZING, AGG.—*Phos.* (compare JAR.)
STRETCHING BODY, AGG.—Thu.
SUPPORTING BREAST, AMEL.—Bell. Cactus. Calc. Carb-an. LAC-CAN. Lyc. Nit-ac. Phos.
TOUCH, AMEL.—CALC.
WALKING, AGG.—Lac-can. Prun. Sep. Stram.

WM. JEFFERSON GUERNSEY, M. D.
4430 FRANKFORD AVENUE, PHILADELPHIA, PA.

